# Simultaneous detection of multiple urinary biomarkers in patients with early-stage diabetic kidney disease using Luminex liquid suspension chip technology

**DOI:** 10.3389/fendo.2024.1443573

**Published:** 2024-08-20

**Authors:** Xinran Li, Xinxin Zhang, Shenglan Wang, Yuan Li, Cheng Meng, Jingyu Wang, Baocheng Chang, Juhong Yang

**Affiliations:** ^1^ National Health Commission (NHC) Key Laboratory of Hormones and Development, Tianjin Key Laboratory of Metabolic Diseases, Chu Hsien-I Memorial Hospital & Tianjin Institute of Endocrinology, Tianjin Medical University, Tianjin, China; ^2^ Department of Endocrinology, Affiliated Hospital of Guangdong Medical University, Zhanjiang, Guangdong, China

**Keywords:** type 2 diabetes mellitus, 24-h urinary albumin excretion rate, estimated glomerular filtration rate, kidney injury molecule-1, retinol-binding protein4, vitamin D binding protein, tumor necrosis factor receptor-2

## Abstract

**Background:**

Several urinary biomarkers have good diagnostic value for diabetic kidney disease (DKD); however, the predictive value is limited with the use of single biomarkers. We investigated the clinical value of Luminex liquid suspension chip detection of several urinary biomarkers simultaneously.

**Methods:**

The study included 737 patients: 585 with diabetes mellitus (DM) and 152 with DKD. Propensity score matching (PSM) of demographic and medical characteristics identified a subset of 78 patients (DM = 39, DKD = 39). Two Luminex liquid suspension chips were used to detect 11 urinary biomarkers according to their molecular weight and concentration. The biomarkers, including cystatin C (CysC), nephrin, epidermal growth factor (EGF), kidney injury molecule-1 (KIM-1), retinol-binding protein4 (RBP4), α1-microglobulin (α1-MG), β2-microglobulin (β2-MG), vitamin D binding protein (VDBP), tissue inhibitor of metalloproteinases-1 (TIMP-1), tumor necrosis factor receptor-1 (TNFR-1), and tumor necrosis factor receptor-2 (TNFR-2) were compared in the DM and DKD groups. The diagnostic values of single biomarkers and various biomarker combinations for early diagnosis of DKD were assessed using receiver operating characteristic (ROC) curve analysis.

**Results:**

Urinary levels of VDBP, RBP4, and KIM-1 were markedly higher in the DKD group than in the DM group (*p* < 0.05), whereas the TIMP-1, TNFR-1, TNFR-2, α1-MG, β2-MG, CysC, nephrin, and EGF levels were not significantly different between the groups. RBP4, KIM-1, TNFR-2, and VDBP reached *p* < 0.01 in univariate analysis and were entered into the final analysis. VDBP had the highest AUC (0.780, *p* < 0.01), followed by RBP4 (0.711, *p* < 0.01), KIM-1 (0.640, *p* = 0.044), and TNFR-2 (0.615, *p* = 0.081). However, a combination of these four urinary biomarkers had the highest AUC (0.812), with a sensitivity of 0.742 and a specificity of 0.760.

**Conclusions:**

The urinary levels of VDBP, RBP4, KIM-1, and TNFR-2 can be detected simultaneously using Luminex liquid suspension chip technology. The combination of these biomarkers, which reflect different mechanisms of kidney damage, had the highest diagnostic value for DKD. However, this finding should be explored further to understand the synergistic effects of these biomarkers.

## 1 Introduction

The annual incidence of type 2 diabetes mellitus (T2DM) is increasing worldwide. Diabetic kidney disease (DKD) is a common complication of DM and the main cause of end-stage renal disease (ESRD) (1, 2). Urinary albumin levels are the traditional standard for diagnosing and classifying DKD; however, increasing evidence indicates that microalbuminuria lacks sensitivity and specificity as a biomarker for the early diagnosis of DKD (3, 4). About 30% patients with microalbuminuria will return back to normal; 30% patients will maintain stable; whereas 30%-40% patients will gradually develop to massive albuminuria, and progress to ESRD even with active treatment (5). Moreover, DKD has heterogeneity and can manifest by albuminuria followed by gradually decline in GFR, or non-proteinuric or non-albuminuric DKD (6, 7). Therefore, there is an urgent need for specific, sensitive, non-invasive biomarkers for the early detection of DKD.

In order to achieve this goal, we need to reexamine the mechanism of the occurrence and development of DKD. Briefly, the pathogenesis of DKD involves interplay of metabolic derangements, glomerular hemodynamic alterations, inflammatory responses and immune dysregulation (8). In the context of diabetes mellitus, hyperglycemia, when combined with hypertension and hyperlipidemia, initiates a cascade of pathological processes. Synergistically, these conditions drive inflammation, promote fibrotic changes, and induce the enlargement of glomeruli—hallmarks of DKD (9).The development and progression of DKD is also influenced by genetic and environmental factors (9). Recently, several urinary biomarkers related to DKD have been identified (10–12). Vitamin D binding protein (VDBP) may serve as a macrophage-activating factor, implicating it in the immune response and the progression of tubulointerstitial fibrosis. Excessive excretion of urinary VDBP suggests tubular dysfunction (13). Similarly, urinary retinol-binding protein4 (RBP4), kidney injury molecule-1 (KIM-1) and cystatin C (CysC) are recognized as highly sensitive markers for tubular dysfunction (14–16). Tumor necrosis factor receptor-1 (TNFR-1) and tumor necrosis factor receptor-2 (TNFR-2) are linked to immune regulation and tissue regeneration, showing a higher specificity for tumor necrosis factor (TNF-α), which are key mediators in the inflammatory response seen in DKD (17, 18). Urinary levels of TNFR-1/2, tissue inhibitor of metalloproteinases-1 (TIMP-1), and epidermal growth factor (EGF) are associated with renal inflammation and fibrosis (19, 20). Nephrin is a podocyte-specific marker, crucial for the integrity of the glomerular filtration barrier, and its dysfunction is implicated in the development of proteinuria, a hallmark of DKD (21). Furthermore, urinary α1-microglobulin (α1-MG) and β2-microglobulin (β2-MG) are established biomarkers for renal tubulointerstitial injury, indicating their utility in monitoring the progression of DKD (22–24). However, the pathogenic mechanisms of DKD are varied and complex; thus, a panel of biomarkers rather than a single biomarker may be a more useful approach for the early detection of DKD (10).Luminex liquid suspension chips are a rapid, accurate, and reliable technique for large-scale testing. They have been widely used in clinical settings with good detection of multiple indicators simultaneously (25, 26). Considering that the dilution ratio, bead region, and biocompatibility for different biomarkers, it is necessary to determine which biomarkers can be detected simultaneously. We assessed 11 urinary biomarkers finally, including α1-MG, β2-MG, RBP4, EGF, KIM-1, VDBP, CysC, nephrin, TNFR-1, TNFR-2, and TIMP-1, to establish a set of sensitive, non-invasive markers that can be detected simultaneously for the early detection of DKD.

## 2 Materials and methods

### 2.1 Participants

This cross-sectional study recruited patients with T2DM admitted to a hospital between February 2019 and February 2020. DM was diagnosed using World Health Organization 1999 criteria (27). All participants provided signed informed consent. The study was conducted according to the principles of the Declaration of Helsinki and was reviewed and approved by the Medical Ethics Committee. Ethics Approval Number: ZXYJNYYkMEC2023–45.

### 2.2 Inclusion and exclusion criteria

Inclusion criteria were T2DM diagnosis, age >18 years, and estimated glomerular filtration rate (eGFR) ≥ 90 mL/min/1.73 m^2^. The exclusion criteria included Type 1 DM (T1DM) or other types of diabetes, acute diabetic complications, such as ketoacidosis, a history of acute kidney injury, urinary calculi, chronic glomerulonephritis, IgA nephropathy, lupus nephritis, polycystic kidney disease, hypertensive nephropathy, gout-associated nephropathy, anemia, neoplasm, severe cardiovascular disease, fever, urinary tract infection, severe hepatic insufficiency or renal insufficiency, and pregnancy. Only individuals with normal kidney function were considered, excluding those with baseline estimated glomerular filtration rate (eGFR) less than 90 mL/min/1.73 m^2^ or macroalbuminuria (24-h urinary albumin excretion rate [UAE] ≥ 300 mg/24 h) (28). Patients were classified as DM or DKD based on their 24-h UAE (DM, < 30 mg/24 h; DKD, 30–300 mg/24 h) (29, 30).

### 2.3 Data collection

Demographic and clinical data obtained from medical records included age, sex, body mass index (BMI), diabetes duration, and blood pressure. Blood samples were drawn from the patients after 8-h overnight fasting. The routine laboratory investigations included serum total cholesterol (TC), triglyceride (TG), high-density lipoprotein cholesterol (HDL-C), low-density lipoprotein cholesterol (LDL-C), creatinine, and uric acid measured using an AU5800 automatic biochemical analyzer (Beckmann Coulter Inc., Brea, CA, USA). Glycated hemoglobin (HbA1c) was measured using an HLC-723G8 HbA1c analyzer (Tosoh Bioscience, Griesheim, Germany). The Chronic Kidney Disease Epidemiology Collaboration equation was used to calculate eGFR. To determine 24-h UAE, 24-h urine was collected for two consecutive days and the mean value was used. Microalbuminuria was measured using immunoturbidimetric assay on Roche Cobas 8000 platform (Roche Cobas c702 module). All specimens were assessed in the Department of Clinical Biochemical laboratory at this hospital.

### 2.4 Luminex liquid suspension chip detection of urinary biomarkers

Early morning midstream urine samples were obtained and preserved at -80°C for 1 year before analysis. Samples were centrifuged at 10,000 rpm for 10 min, and 50 μL of urine supernatant were collected for detection without dilution. Repeated freeze–thaw cycles were avoided.

Luminex liquid suspension chip detection was performed by Wayen Biotechnologies (Shanghai, China) using a human premixed multi-analyte kit according to the manufacturer’s instructions. In brief, urine supernatant obtained by centrifugation was incubated for 2 h in 96-well plates embedded with microbeads, and then incubated with detection antibody for 1 h in darkness. Subsequently, streptavidin-PE was added to each well followed by incubation for 30 min for coloration. Values were read using the Luminex 200 system (Luminex Corporation, Austin, TX, USA). The first chip contained VDBP (LXSAHM-1, dilution 1:1), and the remaining 10 proteins were integrated into another chip (LXSAHM-10, dilution 1:1). The detection range of each protein is shown in the supplementary information.

Specific steps are as follows:

1. Sample Preparation.
  Centrifuge urine samples at 10,000 rpm for 10 minutes, collect the supernatant, and take 50 μL of the original liquid for testing.
2. Standard Preparation.
  Add the required volume of RD6–52 to standard vials as per the manual’s instructions. Mix by inverting several times and incubate on a low-speed shaker for 15 minutes to prepare the standard curve. In this experiment, two repeated tests were set for the standard product. According to the fluorescence detection value (FI) obtained by the Standard product, the Standard Curve and its equation were obtained by fitting the standard curve with multi-parameter mode, and the concentration unit was pg/mL.
3. Chip Detection Operation.
  (1) Sample Incubation: Vortex microbeads at 1,400 rpm for 30 seconds, dilute with RD2–1, vortex again at 1,400 rpm for 30 seconds, and add 50 μL to each well of a 96-well plate. Add 50 μL of the prepared standard curve, samples, and Blank to the corresponding wells, seal with a membrane, and incubate on a plate shaker at 850 rpm for 2 hours at room temperature in the dark.
  (2) Detection Antibody Incubation: Discard the samples and wash with a plate washer three times. Dilute the Biotin Antibody Cocktail with RD2–1 as per the instructions. Add 50 μL of the diluted cocktail to each well, seal, and incubate on a plate shaker at 850 rpm for 1 hour at room temperature in the dark.
  (3) Color Development: Discard the Biotin Antibody Cocktail and wash with a plate washer three times. Dilute Streptavidin-PE with Wash Buffer as per the instructions. Add 50 μL of the diluted Streptavidin-PE to each well, seal, and incubate on a plate shaker at 850 rpm for 30 minutes at room temperature in the dark. Wash with a plate washer three times, resuspend each well with 100 μL of Wash Buffer, seal, and shake at 850 rpm for 2 minutes at room temperature in the dark. Values were read using the Luminex 200 system (Luminex Corporation, Austin, TX, USA).
  (4) Calculate standard curve formula and sample detection concentration: The standard curve formula is used to calculate the sample concentration, which can be used for comparison between samples.

The average Intra-assay coefficient of variation (CV) for technical duplicates of the standard across various markers, including Cystatin C, EGF, Nephrin, RBP4, KIM-1, TIMP-1, TNFR-1, TNFR-2, α1-MG, β2-MG, VDBP, was 2.84%, 1.32%, 1.90%, 2.10%, 2.63%, 2.17%, 1.22%, 1.88%, 2.03%, 1.42% and 2.93%, respectively. The intra-assay CV data fall within an acceptable range (<10%), demonstrating the precision of the assay (31).

### 2.5 Statistical analysis

Quantitative data for normal and non-normal distributions are expressed as mean ± standard deviation and median (first and third quartiles), respectively. For continuous variables, the one-sample Kolmogorov-Smirnov normality test or the Shapiro-Wilk test was used to check the normality of the distribution. Intergroup differences were assessed using independent sample *t*-tests for normally distributed variables and a nonparametric test for non-normally distributed variables. The chi-square test was used to assess categorical data.

To balance differences between the DM and DKD groups, patient characteristics were matched in a 1:1 ratio using propensity score matching (PSM). A total of 13 covariates (sex, age, BMI, DM duration, systolic blood pressure, diastolic blood pressure, HbA1c, eGFR, UA, TC, TG, HDL-C, LDL-C) were included in the PSM model. The caliper width was set to 0.2 of the standard deviation of the logit of propensity score. The balance of covariates after matching was assessed using the standardized difference, with < 10% deemed acceptable.

Spearman’s correlation test was used for all correlation analyses. The area under the curve (AUC), sensitivity, and specificity were calculated as measures of diagnostic accuracy. Receiver operating characteristic (ROC) curve analysis was performed to assess the diagnostic values of the urinary biomarkers. The cut-off value was based on the maximum value of the Youden index. All statistical tests were performed using SPSS software version 22.0 (IBM, Chicago, IL, USA). *P*-values < 0.05 (two-sided) were considered to indicate statistical significance.

## 3 Results

### 3.1 Clinical characteristics of the participants

After propensity matching, a subset of 78 cases was included in the PSM model (DM group, n = 39; DKD group, n = 39). All 13 covariates were evenly matched, and no differences were observed between groups. The average age of the patients was 54.5 years (range, 47–61 years), the average HbA1c level was 8.55% (range, 7.18%–10.13%), and the average BMI was 27.67 ± 3.76 kg/m^2^ (**Table 1**). Among 39 patients with DKD, 18 (46.2%) had diabetic retinopathy (DR); whereas 39 patients with DM did not have DR.

**Table 1:** Clinical characteristics of the study participants.

Characteristics	DM group (*n*=39)	DKD group (*n*=39)	*p*-value
Age	54 (44,63)	55 (50,58)	0.741
Male (n, %)	25 (64)	22 (56)	0.488
BMI (kg/m^2^)	27.15 ± 3.77	28.19 ± 3.73	0.222
SBP (mmHg)	130.46 ± 15.63	133.05 ± 15.13	0.459
DBP (mmHg)	81.77 ± 11.09	83.26 ± 8.51	0.508
DM duration (years)	8 (4,12)	8 (2,13)	0.386
HbA1c (%)	8.1 (7.2,9.8)	8.9 (7.1,10.4)	0.366
FPG (mmol/L)	8.5 (7.1,10)	9.4 (7.0,12.1)	0.463
TG (mmol/L)	1.75 (1.23,2.69)	1.9 (1.65,3.04)	0.118
TC (mmol/L)	4.98 ± 1.07	5.31 ± 1.30	0.229
HDL-C (mmol/L)	1.1 (1.02,1.21)	1.13 (0.96,1.38)	0.539
LDL-C (mmol/L)	3.54 ± 0.93	3.42 ± 0.88	0.557
BUN (mmol/L)	4.99 (4.27,5.94)	5.01 (4.38,6.54)	0.579
SCr (μmol/L)	63.81 ± 10.76	59.51 ± 13.21	0.119
eGFR (ml/min/1.73 m^2^)	103.31 ± 7.5	106.08 ± 12.46	0.239
SUA (μmol/L)	301.8 (250.5, 370.3)	328.6 (249.7, 392.1)	0.433
24-h UAE (mg/24 h)	11.7 (8.87, 17.05)	78.89 (47.19,135.84)	<0.001
diabetic retinopathy (n, %)	0	18 (46.15%)	–

BMI, body mass index; SBP, Systolic blood pressure; DBP, diastolic blood pressure; HbA1c, glycated hemoglobin; FPG, fasting plasma glucose; TG, triglyceride; TC, total cholesterol; HDL-C, high-density lipoprotein cholesterol; LDL-C, low-density lipoprotein cholesterol; BUN, blood urea nitrogen; SUA, serum uric acid; SCr, serum creatinine; eGFR, estimated glomerular filtration rate; 24-h UAE, 24-hour urinary albumin excretion.

### 3.2 Comparison of urinary biomarkers between groups

Baseline data were well-balanced after matching. The 11 urinary biomarkers were accurately detected in all 78 patients in the DM and DKD groups (**Table 2**). Data not within the scope of the test were excluded. The VDBP, RBP4, and KIM-1 levels were markedly higher in the DKD group than in the DM group (*p*<0.05). We found no between-group differences in the CysC (*p* = 0.146), nephrin (*p* = 0.289), EGF (*p =* 0.330), α1-MG (*p =* 0.415), β2-MG (*p =* 0.585), TIMP-1 (*p =* 0.138), TNFR-1 (*p =* 0.133), or TNFR-2 (*p =* 0.081) values.

**Table 2:** Comparison of different urine biomarkers between DM group and DKD group.

Biomarkers	DM group	DKD group	*p*-value
CysC	11.71 (1.56,19.9) (n=38)	13.25 (3.64,30.95) (n=39)	0.146
nephrin	1.94 (0.25,6.62) (n=39)	2.46 (0.8,5.8) (n=39)	0.289
EGF	7.82 (3.02,9.94) (n=39)	8.46 (5.5,9.09) (n=39)	0.330
KIM-1	0.12 (0.04,0.53) (n=33)	0.48 (0.16,0.92) (n=38)	0.044
RBP4	30 (7,60) (n=31)	73 (29,130) (n=32)	0.004
α1-MG	6750 (4410,9220) (n=39)	7130 (4860,10210) (n=39)	0.415
β2-MG	2.47 (0.18,4.19) (n=15)	2.46 (0.41,8.65) (n=14)	0.585
VDBP	35 (12,61) (n=39)	146 (52,239) (n=39)	<0.001
TIMP-1	0.061 (0.024,0.232) (n=35)	0.11 (0.039,0.378) (n=37)	0.138
TNFR-1	0.87 (0.29,1.50) (n=39)	1.3 (0.74,1.81) (n=39)	0.133
TNFR-2	1.71 (0.39,6.37) (n=39)	4.35 (1.61,7.54) (n=39)	0.081

CysC, Cystatin C; EGF, epidermal growth factor; KIM-1, kidney injury molecule-1; RBP4, retinol-binding protein4; α1-MG, α1-microglobulin; β2-MG, β2-microglobulin; VDBP, vitamin D binding protein; TIMP-1, tissue inhibitor of metalloproteinases-1; TNFR-1, tumor necrosis factor receptor-1; TNFR-2, tumor necrosis factor receptor-2.

Data outside of the detection range were not recorded. All standards are expressed in units of ng/mL.

### 3.3 Urinary biomarker correlations with 24-h UAE

The RBP4 (*r* = 0.351; *p* = 0.005), KIM-1 (*r* = 0.319; *p* = 0.007), TNFR-2 (*r* = 0.236; *p* = 0.038), and VDBP (*r* = 0.462; *p* < 0.001) urinary biomarkers were significantly positively correlated with 24-h UAE in 78 cases (**Table 3**).

**Table 3:** Correlation analysis of urine biomarkers and 24-h UAE in 78 patients.

		RBP4	KIM-1	TNFR-2	VDBP
24-h UAE	*r* value	0.351	0.319	0.236	0.462
	*p* value	0.005	0.007	0.038	<0.001

RBP4, retinol-binding protein4; KIM-1, kidney injury molecule-1; TNFR-2, tumor necrosis factor receptor-2; VDBP, vitamin D binding protein. R value refers to spearman’s correlation coefficients.

### 3.4 Predictive value of individual and combined urinary biomarkers

**Figure 1:**
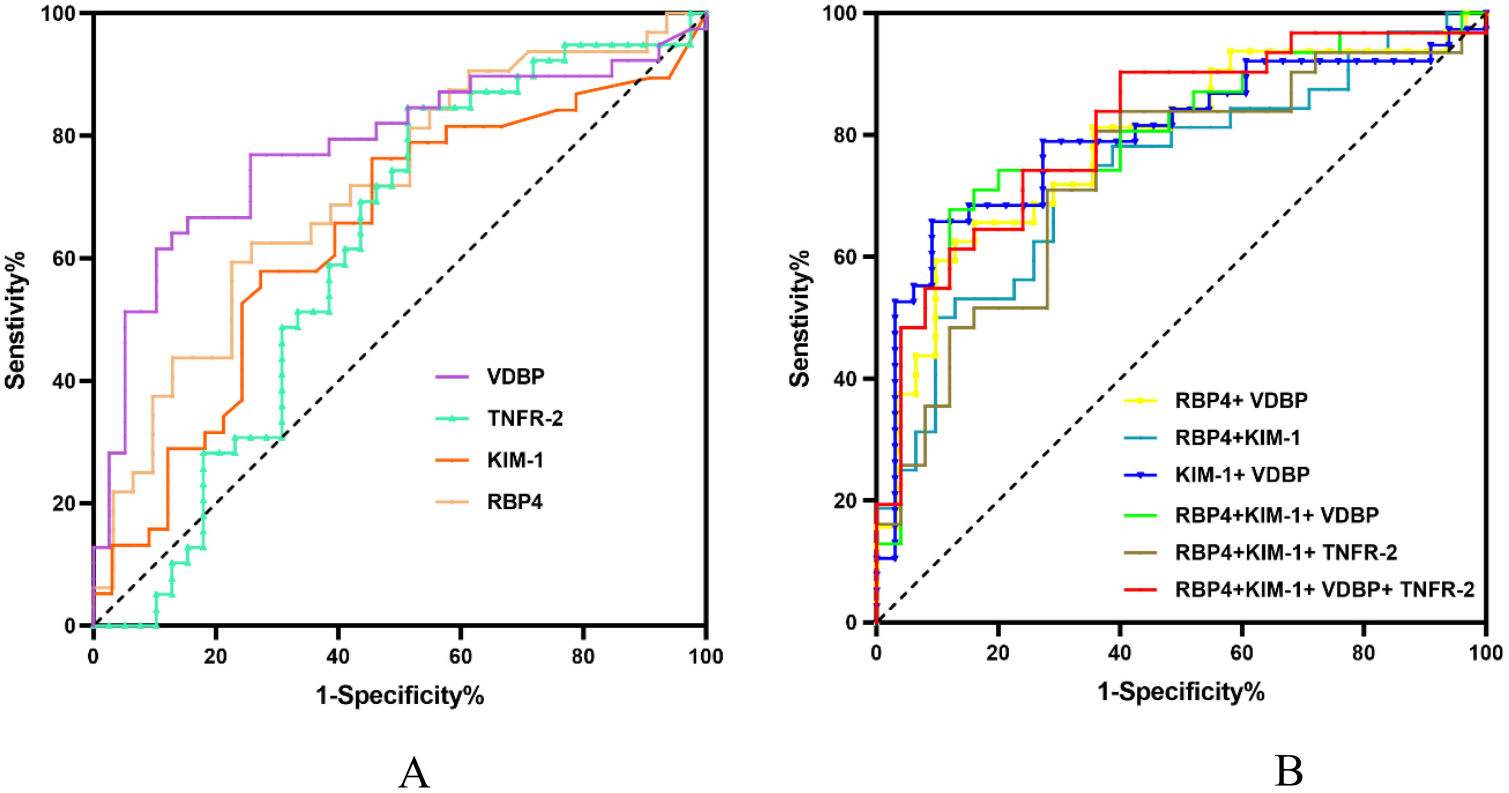
ROC curves of different biomarkers for the diagnosis of DKD. AUC, area under the curve; DKD, diabetic kidney disease; ROC, receiver operating characteristic. **(A)** ROC curves of a single urinary biomarker. **(B)** ROC curves of different combinations of urinary biomarkers.

RBP4, KIM-1, TNFR-2, and VDBP reached *p* < 0.1 in univariate analysis and were entered into in the final analysis. The diagnostic values of the biomarkers were assessed individually and in combination by AUC, sensitivity, and specificity scores (**Table 4**). VDBP had the highest AUC (0.780, *p* < 0.01), followed by RBP4 (0.711, *p* < 0.01), KIM-1 (0.640, *p* = 0.044), and TNFR-2 (0.615, *p* = 0.081). The cut-off values were 98.36 for VDBP (sensitivity, 64.1%; specificity, 87.2%), 61.29 for RBP4 (sensitivity, 76.3%; specificity, 54.5%), 0.16 for KIM-1 (sensitivity, 59.4%; specificity, 77.4%), and 1.27 for TNFR-2 (sensitivity, 84.6%; specificity, 48.7%). Further analysis of the predictive performance of various biomarker combinations revealed that the combined AUC of RBP4, KIM-1, TNFR-2, and VDBP was 0.812 (*p* < 0.01), which was higher than all other combinations (**Table 4**, **Figure 1**).

**Table 4:** Evaluation of urinary markers and different combinations in the diagnosis of DKD.

Items	AUC (95%CI)	Cut-off value (ng/mL)	Sensitivity (95%CI)	Specificity (95%CI)
RBP4	0.711 (0.584–0.839)	61.29	0.763 (0.61–0.87)	0.545 (0.38–0.70)
KIM-1	0.640 (0.508–0.771)	0.16	0.594 (0.42–0.74)	0.774 (0.60–0.89)
TNFR-2	0.615 (0.486–0.743)	1.27	0.846 (0.70–0.93)	0.487 (0.33–0.64)
VDBP	0.780 (0.672–0.887)	98.36	0.641 (0.48–0.77)	0.872 (0.73–0.94)
RBP4+KIM-1	0.739 (0.615–0.862)		0.719 (0.55–0.84)	0.710 (0.53–0.84)
RBP4+ VDBP	0.787 (0.673–0.902)		0.594 (0.42–0.74)	0.903 (0.75–0.96)
KIM-1+ VDBP	0.798 (0.691–0.905)		0.658 (0.50–0.79)	0.909 (0.76–0.97)
RBP4+KIM-1 + VDBP	0.800 (0.682–0.918)		0.677 (0.50–0.81)	0.880 (0.70–0.96)
RBP4+KIM-1 +TNFR-2	0.735 (0.602–0.868)		0.806 (0.64–0.91)	0.640 (0.45–0.80)
RBP4+KIM-1 +VDBP+TNFR-2	0.812 (0.698–0.925)		0.742 (0.57–0.83)	0.760 (0.57–0.89)

combination1, RBP4+KIM-1; combination2, RBP4+VDBP; combination3, KIM-1+ VDBP; combination4, RBP4+KIM-1+ VDBP; combination5, RBP4+KIM-1+TNFR-2; combination6, RBP4+KIM-1+VDBP+TNFR-2.

## 4 Discussion

DKD is one of the most prevalent microvascular complications of T1DM and T2DM, occurring in 25–40% of patients with DM (32). DKD is a common cause of ESRD and an independent risk factor for all-cause and cardiovascular mortality in patients with DM (32), resulting in a significant global health and socioeconomic burden (33). A kidney biopsy is essential for accurately diagnosing DKD versus NDKD, but its invasive nature, cost, and patient reluctance restrict its clinical application. Retrospective kidney biopsy studies in type 2 diabetes patients reveal several clinical features that distinguish DKD from other kidney conditions (34). Albuminuria as a traditional indicator for DKD has limited diagnostic sensitivity and specificity (3, 4). About 30% patients with microalbuminuria will return back to normal; 30% patients will maintain stable; whereas 30%-40% patients will gradually develop to massive albuminuria, and progress to ESRD even with active treatment (5). Moreover, DKD has heterogeneity and can manefest by albuminuria followed by gradually decline in GFR, or non-proteinuric or non-albuminuric DKD (6, 7). Although a lot of urinary biomarkers related to DKD have been identified recently, a single biological marker is unlikely to be specific or sensitive enough for good predictive value (10–12). Therefore, a panel of biomarkers may be a better approach for the early detection of DKD (10). The study selected 11 biomarkers closely related to kidney disease, which may be used for the early prediction of DKD and cover various DKD mechanisms (11, 35). Additionally, considering biocompatibility, dilution ratio, and bead region, we excluded alternative markers like fetuin-A, and the final selection of 10 biomarkers can technically be detected on a chip simultaneously (https://www.rndsystems.com/cn/products/human-luminex-discovery-assay_lxsahm). In this study we used Luminex liquid suspension chip to systematically assess the predictive value of 11 urinary biomarkers for DKD and to identify a set of sensitive, non-invasive urinary biomarkers to be measured simultaneously for the early detection of DKD.

We found that the urinary levels of VDBP, RBP4, and KIM-1 were significantly higher in the DKD group than in the DM group. Moreover, we found a positive association between the urinary VDBP, RBP4, and KIM-1 levels and 24-h UAE. The baseline characteristics and blood biochemical indices of the DM and DKD groups were matched using PSM to reduce the influence of confounding variables and selection bias. We excluded patients with non-DKD, and only patients with microalbuminuria were included in the study to more accurately reflect early-stage DKD. We found that VDBP, RBP4, and KIM-1 were early markers of DKD.

Assessment of VDBP, RBP4, and KIM-1 individually revealed that VDBP had the highest diagnostic value for DKD. ROC analysis revealed that the optimal cut-off value for detecting DKD was 98.36 ng/mL, corresponding to 64.1% sensitivity and 87.2% specificity, suggesting that VDBP may be a reliable biomarker for screening patients with DKD. VDBP, a 58-kDa glycoprotein, is the main carrier protein for circulating vitamin D and its metabolites. The complex formed by VDBP and 25-hydroxyvitamin D (25OHD) can be actively recovered by receptor endocytosis mediated by megalin after glomerular filtration (36). A previous study found that higher 25OHD values were significantly correlated with a lower risk for albuminuria after adjusting for confounding variables (37). In our study, VDBP was significantly positively correlated with urinary albumin, which may be related to low 25OHD levels. In addition to binding and transporting vitamin D and its metabolic products, VDBP may act as a macrophage-activating factor and participate in the immune response and tubulointerstitial fibrosis (38, 39). Excessive excretion of urinary VDBP may indicate tubular dysfunction; several clinical studies have suggested that urinary VDBP is a potential biomarker for the early prediction and detection of DKD (39–41). Our findings are consistent with those of previous studies. Nevertheless, the specificity of VDBP as a predictive biomarker should be considered if used for the early prevention of DKD (38).

We found that urinary RBP4 and KIM-1 are sensitive markers for early renal damage. Urinary RBP4 is a highly sensitive marker for tubular dysfunction (14, 15). Elevated urinary RBP4 is caused by a decrease in RBP4 reabsorption in the proximal renal tubule, which is mediated by the megalin-cubilin receptor complex (42). Our previous study showed that urinary RBP is a reliable and better predictor of DKD than transferrin, immunoglobulin G, β-galactosidase, or β2-MG (43). As a specific and sensitive biomarker for proximal tubule damage, KIM-1 phagocytizes apoptotic cells and remodels injured epithelial cells (16). Inflammation and oxidative stress are the key contributors to kidney injury in DM (44). Our finding that KIM-1 is an independent marker for early DKD is consistent with results of previous studies (45–47). TNFR-2 is related to immune regulation and tissue regeneration and has a higher specificity for tumor necrosis factor (TNF-α) (17). Previous studies have shown an association between plasma TNFRs and a decline in renal function in DKD (48–50). However, urinary TNFR has not been studied as richly as plasma TNFR as a biomarker for DKD. We found that although urinary TNFR-2 levels were not significantly different between the DKD and DM groups, adding TNFR-2 to the model improved the prediction of DKD. It may be that increased urinary TNFR-2 excretion reflects increased shedding of TNFR-2 from cell membranes in response to TNF-α and increased circulating TNFR-2. Thus, TNFR-2 levels may be an important clinical predictor of DKD onset.

The pathogenic mechanisms of DKD are complex and driven by a series of maladaptive metabolic, hemodynamic, inflammatory, and fibrotic processes (8). Given the complexity of the pathogenic mechanisms of DKD, a single biomarker may be a less specific and sensitive predictor of DKD than a panel of biomarkers derived from multiple pathophysiological processes. Four biomarkers (RBP4, KIM-1, TNFR-2, and VDBP) that reached *p* < 0.1 in univariate analysis were included in the final analysis. Of those, VDBP had the highest AUC (0.780, *p* < 0.01), followed by RBP4 (0.711, *p* < 0.01), KIM-1 (0.640, *p* = 0.044), and TNFR-2 (0.615, *p* = 0.081). However, the combination of the four urinary biomarkers had the highest AUC (0.812), with a sensitivity of 0.742 and a specificity of 0.760. Because VDBP, TNFR-2, RBP4, and KIM-1 have similar molecular weights and concentrations, they can be detected simultaneously using a Luminex liquid suspension chip. The combination of these four biomarkers provided the best predictive value for the diagnosis of DKD; moreover, the technique is feasible for direct clinical application.

In our study, CysC, nephrin, α1-MG, β2-MG, EGF, TNFR-1, and TIMP-1 did not significantly impact DKD in the adjusted PSM groups. Urinary TNFR-1, TIMP-1, and EGF are associated with renal inflammation and fibrosis. Diabetic renal fibrosis is an irreversible pathological change in the late stage of DKD (51). We found that urinary TNFR-1, TIMP-1, and EGF values were not effective early predictors of DKD. TNFR-1 is present mainly in glomerular and tubular endothelial cells, and high serum levels of TNFR-1 are associated with interstitial inflammation and fibrosis (18). To our knowledge, no previous study has identified urinary TNFR-1 as a biomarker for DKD. TIMP-1 expression is universally upregulated in experimental kidney disease along with increased interstitial fibrosis (19). A previous study found a marked increase in urinary TIMP-1 in patients with DM associated with the severity of diffuse glomerulosclerosis (52). A German study found that urinary TIMP-1 levels were increased in patients with chronic kidney disease with varying degrees of renal impairment (53). Another study found that TIMP-1 levels did not increase in the early stage of renal injury in children with T1DM (54). Although a Spanish study found that serum TIMP-1 levels were increased in the early stages of DKD, the authors did not measure urinary TIMP-1 and only included patients with GFRs < 60 mL/min/1.73 m^2^ (55). EGF, a small peptide growth factor, is produced primarily in the ascending portion of Henle’s loop and the distal tubule of the kidney (56). Renal biopsy-based studies have shown that lower urinary EGF levels are significantly associated with increased tubular atrophy and interstitial fibrosis (20). Urinary EGF may reflect a rapid decline of renal function in early DKD (57). Boris B.Betz reported that whereas a greater proportion of patients in the highest tertile of uEGF had an ACR greater than 0.5 mg/mmol; however, because potential confounding factors were not adjusted, the authors were unable to conclude that urinary EGF was associated with albuminuria. Therefore, neither urinary TNFR-1 nor TIMP-1 nor EGF could be used as good predictors of early stage of DKD based on the current understanding.

Nephrin is a podocyte-specific marker (58). Urinary nephrin mRNA expression may increase before the development of microalbuminuria, reflecting early podocyte damage in patients with DM. A previous study found that nephrinuria was associated with low eGFR in patients with normoalbuminuria DKD. The authors used Western blot assay to detect urinary nephrin fragments rather than a quantitative approach (59, 60). Another study found no association between urinary nephrin and albuminuria in DKD (61). In summary, our findings indicate that urinary nephrin is not a good predictor of early renal dysfunction. In patients with T2DM, serum CysC is a more sensitive marker of early renal impairment than creatinine. CysC is a 13-kDa low-molecular-weight protein that is almost completely reabsorbed and catabolized in the proximal tubules after being freely filtered through the glomeruli. Elevated urinary CysC levels reflect renal tubular dysfunction in various nephropathies. However, few studies have investigated the correlation between urinary CysC and early DKD. Future clinical studies are needed to validate the predictive value of urinary CysC for DKD.

Urinary α1-MG and β2-MG are classic biomarkers for renal tubulointerstitial injury in DKD (22, 23, 62). They reflect the renal tubular reabsorption capacity in patients with DM (10). A previous study found that α1-MG was related to the duration, severity, and control of DM (22). We corrected for the duration and severity of DM, and as our participants were in the early stage of DKD, we found no significant differences in urinary α1-MG and β2-MG. Moreover, reports of differences in urine β2-MG levels in patients with DM and controls are conflicting, as is the role of urine β2-MG in detecting early renal injury (10). These disparities may be related to changes in the urine pH. β2-MG is unstable and undergoes degradation at room temperature when the pH is below 5.5, or at body temperature when the pH is below 6.0 (63). Given its instability, β2-MG is not useful for medical diagnoses (10, 63).

Our study had several limitations. First, we used a cross-sectional design with a small sample size. Although the baseline characteristics and blood biochemical indices were matched using PSM to reduce the influence of confounding variables and selection bias, a prospective study with a large sample size is needed to confirm the causal relationships between RBP4, KIM-1, TNFR-2, VDBP, and DKD. Second, as all patients were recruited from an urban area, China, our findings cannot be extrapolated to other populations in other regions. Third, recent studies have identified potential DKD biomarkers, such as Neutrophil gelatinase-associated lipocalin (NGAL) for tubular injury and fetuin-A, indicating filtration barrier disruptions and impaired tubular uptake. Other emerging biomarkers such as microvesicles, urinary exosomes, and microRNAs are also being explored (35, 64). A subset of biomarkers were selected in this study, and other meaningful indicators may be missed. This practice has selection bias, which needs to be confirmed by more studies in the future. Additionally, the patients in this study had poor blood sugar control, which may have had some potential impact on the results because blood glucose levels affect these markers to varying degrees. Future studies involving patients with stable glucose and normal populations are needed.

The Luminex technology currently faces challenges, primarily high costs related to equipment, reagents, and maintenance, which can restrict its use in settings with limited resources. Additionally, there are limitations in multiplexing due to a finite number of fluorescence channels and potential interference. To improve, we can achieve economies of scale by bulk purchasing reagents or seek funding to reduce initial costs. To enhance multiplexing, we can employ markers with distinct fluorescence profiles to minimize channel crosstalk and develop sophisticated algorithms to discern closely spaced fluorescence signals. These adjustments can make Luminex technology more cost-effective and improve the efficiency and precision of multiplex assays. Moving forward, we intend to extend the application of our research methodologies to the analysis of urinary and serum specimens obtained from individuals exhibiting a precipitous decline in glomerular filtration rate (GFR), as well as those with non-proteinuric or non-albuminuric forms of diabetic kidney disease (DKD), thereby augmenting the precision of our diagnostic assays. Additionally, we aim to investigate the potential utility of extending these methods to oncology, hematology, etc.

In summary, we used a Luminex liquid suspension chip technology to assess the predictive value of 11 urinary biomarkers reflecting glomerular injury, tubular injury, immunity, and inflammation for the diagnosis of DKD. We found that the simultaneous detection of urinary VDBP, TNFR-2, RBP4 and KIM-1 in patients with T2DM improved diagnostic accuracy for early DKD. This finding needs to be further explored to understand the synergies of these biomarkers.

## Data Availability

The raw data supporting the conclusions of this article will be made available by the authors, without undue reservation.
