# Supplementary material for: Simultaneous detection of multiple urinary biomarkers in patients with early-stage diabetic kidney disease using Luminex liquid suspension chip technology
Source: Front Endocrinol (Lausanne). 2024 Aug 20;15:1443573. doi: 10.3389/fendo.2024.1443573 (PMC11369644; doi:10.3389/fendo.2024.1443573)
Supplement: Supplementary file 1 [file Table1.docx]

**Supplementary data**

Chip model and detection range

| Chip model | biomarker | detection range (pg/mL) |
| --- | --- | --- |
| LXSAHM-10 | Cystatin C | 185.79~135440 |
|  | EGF | 2.54~1850 |
|  | Nephrin | 157.09~114520 |
|  | RBP4 | 191.58~139660 |
|  | KIM-1 | 25.53~18610 |
|  | TIMP-1 | 14.61~10650 |
|  | TNFR-1 | 16.5~12030 |
|  | TNFR-2 | 3.24~2360 |
|  | α1-MG | 41122.28~29978000 |
|  | β2-MG | 28.07~20460 |
| LXSAHM-1 | VDBP | 2700.62~1968800 |

**Supplementary Table1** Chip model and detection range for each marker. CysC, Cystatin C; RBP, retinol-binding protein; EGF, epidermal growth factor; KIM-1, kidney injury molecule-1; TIMP-1, tissue inhibitor of metalloproteinases-1; TNFR-1, tumor necrosis factor receptor-1; TNFR-2, tumor necrosis factor receptor-2; α1-MG, α1-microglobulin; β2-MG, β2-microglobulin; VDBP, vitamin D binding protein

Comparison of different characteristics between DM group and DKD group before propensity score matching

| Characteristics | DM group（*n*=585） | DKD group（*n*=152） | *p*-value |
| --- | --- | --- | --- |
| Age | 53.84±9.42 | 52.81±0.78 | 0.229 |
| Male (n, %) | 334 (57.1) | 89 (58.6) | 0.746 |
| DM duration (years) | 9 (3, 13) | 8 (3, 13) | 0.953 |
| BMI (kg/m^2^) | 26.32±3.71 | 27.92±4 | ＜0.001 |
| SBP (mmHg) | 130.48±14.11 | 136.38±15.68 | ＜0.001 |
| DBP (mmHg) | 79.91±9.49 | 83.44±9.53 | ＜0.001 |
| HbA1c (%) | 8.44±2.01 | 8.84±1.7 | 0.015 |
| TG (mmol/L) | 1.62 (1.04, 2.44) | 1.82 (1.23, 2.77) | 0.007 |
| TC (mmol/L) | 5.15±1.11 | 5.15±1.11 | 0.968 |
| HDL-C (mmol/L) | 1.21±0.37 | 1.11±0.24 | 0.003 |
| LDL-C (mmol/L) | 3.37±0.92 | 3.36±0.89 | 0.927 |
| BUN (mmol/L) | 5.27±1.2 | 5.44±1.18 | 0.125 |
| eGFR(ml/min/1.73 m^2^) | 102.94 (97.56, 110.2) | 103.78 (99.01, 111.4) | 0.202 |
| SCr (μmol/L) | 60.74±11.44 | 60.2±12.13 | 0.613 |
| SUA (μmol/L) | 304.02±79.73 | 314.85±74.21 | 0.131 |
| 24-h UAE (mg/24 h) | 12.5（7.69,16.8） | 68.25 (41.77, 138.31) | ＜0.001 |

**Supplementary Table2** BMI, body mass index; SBP, Systolic blood pressure; DBP, diastolic blood pressure; HbA1c, glycated hemoglobin; TG, triglyceride; TC, total cholesterol; HDL-C, high-density lipoprotein cholesterol; LDL-C, low-density lipoprotein cholesterol; BUN, blood urea nitrogen; SUA, serum uric acid; SCr, serum creatinine; eGFR, estimated glomerular filtration rate; 24-h UAE, 24-hour urinary albumin excretion
